# Ethical Decision-Making in Humanitarian Medicine: How Best to Prepare?

**DOI:** 10.1017/dmp.2020.85

**Published:** 2021-08

**Authors:** Kadri Simm

**Affiliations:** Institute of Philosophy and Semiotics, University of Tartu, Tartu, Estonia.

**Keywords:** ethics training, disaster bioethics, disaster response, moral distress, value-reflection

## Abstract

Ethical decision-making during humanitarian medical response is a topic of great moral as well as practical importance. The context of humanitarian disasters, often characterized by acute time-pressure, lack of resources, the unfamiliarity of circumstances, is stressful for medical professionals. The overall aim of this article is pragmatic, to introduce briefly the importance and context for preparing medical disaster response personnel for ethical decision-making and then to provide a discussion case and explain the particular value-reflection methodology. The focus of methodology is on providing space for the emotional and stressful aspects of ethics training for disasters.

## ETHICAL DECISION-MAKING IN HUMANITARIAN MEDICINE: HOW BEST TO PREPARE?

The need to prepare people for stressful ethical decision-making before they are flown into humanitarian zones is widely acknowledged. For routine medical dilemmas, best practice guidelines outline the preferred way of action, for example by insisting that certain moral principles outweigh others. But disaster contexts are complicated and often people need to make difficult decisions that can haunt them later on (even though they might still believe that they did the right thing). This may result in feelings of regret and powerlessness, team conflicts, frustration, as well as in physical symptoms (burnout, pains) and illness. The overall aim of this article is pragmatic, to introduce briefly the context for preparing medical disaster response personnel for ethical decision-making, to provide a discussion case and explain the methodology.

### Moral Distress

Context of humanitarian disasters, often characterized by acute time-pressure, lack of resources, the unfamiliarity of circumstances, is stressful for medical (as well as other) professionals. Stress has been shown to have a negative impact on decision-making capacity and consistency.^1^ Decisions need to be made, but the ethical principles and practices of traditional clinical care might not always be helpful. Rather the opposite, these might constitute a source of additional distress as the applicability of individual-centered patient care (focusing on patient autonomy and relying on the availability of numerous care options) or the home-country standard of care, becomes questionable.

Moral distress has been most systematically studied in nursing, including the development of measurements of moral distress,^2,3^ various stress-management tools and programs^4^ as well as numerous qualitative and quantitative studies. Moral distress has sometimes been used in humanitarian ethics context^5^ in addition to synonymous notions like ethical challenges or moral fatigue. Cynda Rushton has described the phenomenon as follows: “Moral distress is really about the anguish that clinicians often feel when they are confronted with situations where their integrity is being compromised. /./ The consequence of that is often a very profound sense of anguish and suffering and a sense of violation of their sense of being a good nurse, a good doctor or whatever their role is”.^6^

Well-known is Andrew Jameton’s conceptualization of moral anguish into three distinct categories: moral distress, moral dilemma, and moral uncertainty. While the first of these has received the most attention in nursing literature, all three are highly relevant for humanitarian healthcare context. Jameton defined *moral distress* as “the painful psychological disequilibrium that results from recognizing the ethically appropriate action, yet not taking it, because of such obstacles as lack of time, supervisory reluctance, an inhibiting medical power structure, institution policy, or legal considerations”.^7^ *Moral dilemma* concerns a situation where the right course of action is not known and all possible options involve conflicts between different moral principles (thus all solutions in dilemma cases are such that they involve the breaking of some important principle or value). For example, choosing between attending to one very severely injured patient or to two slightly less severely injured patients is a dilemma when you can do only one of those actions (you cannot attend to all three). Any way you choose to act, there is a failure in upholding a certain moral principle or value. The third type of anguish, *moral uncertainty*, pertains to situations where one is unsure what moral principles, values, or rules to apply. While this categorization of moral anguish is interesting and useful, it is not clear that these types exist distinctly in the context of humanitarian medical care. Rather, in humanitarian crisis, a medical professional is likely to encounter all of those types of moral anguish as well as their various combinations.

Studies of physicians and nurses, especially in critical care contexts, have associated moral distress with higher turnover intention rates.^8^ The fact that disaster medicine is highly stressful is illustrated by the fact that less than half of recruits choose to participate in their second assignment.^9^ The reasons for this are various, for example, it might well be that recruits want to experience such missions once but have no long-term engagement plans. Yet, it is obvious that assignments can be very demanding, postdeployment there is an increased risk for depression and burnout,^10^ and in addition to many other challenges (logistics, lack of sleep, security issues, etc.), the moral decision-making distress is often present. Humanitarian disaster context is well known for the tragic choices and compromises involved.

Schwartz et al. have identified the following four broad categories of ethical challenges for humanitarian medical workers^11^: (1) resource scarcity and associated allocation issues in the context of acute and widespread needs; (2) historical, political, social, cultural, and other circumstances; (3) aid agency policies that are seen as pressurizing and constraining necessary action; and (4) divergent expectations around medical professionals’ roles and duties.

The danger of feeling and being complicit in some wrongdoing is one that humanitarian aid workers are very likely to encounter.^12^ From the perspective of ethical regulation, humanitarian disasters can often be a site for clashing ethical frameworks: patient autonomy and interests are central for everyday clinical practice, yet the epidemiological and other public health concerns can often trump these principles in disaster settings. In disasters, humanitarian medical professionals will likely make decisions that they would not make in their everyday jobs and that can be an important source of moral distress. The dangers of relativism are apparent here but so are the perils of blindly applied universalist thinking.

### Can People Be Better Prepared?

For decades humanitarian medical organizations have prepared medical professionals for assignments in disaster zones. On top of the medical training, it is increasingly common to provide instruction also on various “soft” aspects of disaster response, that is, cultural and religious contexts as well as ethical decision-making, teambuilding, conflict resolution, and media communication skills. Disaster preparedness literature uses extensively the language of competencies to describe the knowledge and skills most needed,^13^ yet ethical competencies are rarely raised. But among the scarce resources of the disaster setting, the capacity for ethical decision-making can constitute a significant resource in itself, especially given the fact how the neglect of ethical issues can generate serious problems (erosion of trust, increased moral distress of all involved, stigmatization, etc.).^14^

A successful training could contribute to better psychological coping and stronger resilience for returning healthcare staff. People would be better prepared and more aware of the inevitable ethical challenges that arise in such contexts. In the long term, this could also mean that more people are willing to continue with humanitarian aid work and levels of moral distress, widely understood, are kept under control.

What is the best way to achieve these results? Learning about professional codes, available decision-making tools, guidelines, and best practice guides is very important and often helpful. But even quite practical and pragmatic cases and guidelines often do not engage with the emotional side of moral decision-making. After all, you might have acted ethically correctly but you will still feel stressed because you also abandoned certain important moral values. This is especially so if decision-making involves the so-called tragic choices where the “right thing to do” is context-dependent, involves choosing between incommensurable principles, and its legitimation depends on the quality of the reflection. My argument is that ethics teaching needs to better incorporate the contextual and emotional aspects of ethical decision-making.^15^ (The idea that emotions are heavily involved in ethics and that reasonable decisions need to be accompanied by appropriate emotions goes back to Aristotle and David Hume. More recently moral psychologists have studied how people actually make ethical choices and concluded that “moral intuitions come first and directly cause moral judgements”.^15^)

Training should take account of the fact that ethical decision-making is always complex, involving not only knowledge of guidelines and principles but also social, organizational, and emotional components. Could a more deliberate engagement with emotions and intuitions in ethics training support decision-making in difficult disaster context and contribute to lowering moral distress? What practical aspects should such ethics training take into account to better prepare people for ethical dilemmas and support the role of reasoning?

#### Reasoning With Others

Morality is a social and intersubjective phenomenon; we feel the need to relate and justify our moral decisions to others. Thinking on our own, we rarely argue ourselves out of our initial judgments and tend to accept evidence that supports our existing views but the “social persuasion” aspect of morality (reasoning with and through others) has been shown to increase the causal role of moral reasoning.^16^ There is apparently an evolutionary basis for our tendencies to harmonize our judgments with others, our “moral machinery” was not designed for accuracy but harmony with our peers. This, of course, can work both ways, as group-think and its various negative consequences have demonstrated. For effective ethics teaching where reasons and arguments can play a role, this means more room for joint discussions. This is especially relevant in humanitarian context where most work (including ethical deliberation) is done in teams.

#### Early Preparation

Moral reasoning tends to be ineffective in conflict situations where all sides are already convinced and settled into their positions. Reasons and facts are more likely to have an effect when people have not yet formulated their position and there is little time pressure to decide quickly. Ethics training before a humanitarian assignment allows for a more unhurried engagement with ethical reasoning.

#### Learning the Right Words

As our moral views are often anchored in intuitions or “a sense” of something being right or wrong, ethics training can provide us with the vocabulary of ethical thinking (central concepts, important values, and principles). We can then give voice to those gut feelings and better articulate and negotiate our concerns.

#### Attention to the Organizational Context

Although some ethics theories claim that context does not or should not matter to ethical practice, empirical research has shown that it does. From organizational ethics we know that many professional and research ethics scandals ought not be explained by means of references to the evil wrongdoing of one person (the so-called “bad apple”) but misconduct usually becomes possible because of failures and flawed practices within a particular organizational culture. While ethics training cannot take responsibility for guaranteeing a context that supports integrity and makes ethical practice feasible, it can provide information about the availability of guidelines, best practice schemes and other established procedures that operate in many humanitarian organizations. The objective of those documents and practices is precisely to manage the context and facilitate ethical decision-making.

#### Cases, Cases, Cases

It is difficult, if not impossible, to teach emotions and intuitions, but there is something to be said for mimicking situations, for putting yourself in the shoes of another, and experiencing the stress of disagreement (even though we know it is “just” a training). Films and literary works are increasingly being used in medical education to provide a more nuanced and holistic learning opportunity. Case-based methodology (there are many), is by now a staple in most applied ethics training, and it allows people to discuss and reflect upon these issues and upon their intuitions. We have developed a method that targets the following aspects of ethical decision-making: taking of individual responsibility, recognition of and dealing with peer disagreement, teamwork and consensus-building, and moral stress associated with tragic choices.

### Value-Reflection Methodology

Below I will illustrate how these different elements of moral reflection come together in a case-based value-reflection approach that we have developed in University of Tartu Centre for Ethics.^17^ The example case focuses on female genital mutilation (FGM), but the method can accommodate various topics: cases where the patient-centered care conflicts with the requirements of public health in the context of resource-scarcity, the dilemmas of conducting research in a disaster aftermath, or situations where colleague’s low tolerance of stress might negatively affect care-provision.

The case is introduced (best in written form or projected, see Table 1, Step 1. Case Description).^18^ Group size should allow for meaningful discussion (ideally 5-6). When usually in case-based training participants will have to come up with their response, we have prepared a choice of options to choose from (Table 1, Step 2. Options). The reasons for this are that the options are designed in a way that excludes an easy or obviously right action, thus forcing participants into emotionally uncomfortable territory. Each participant has to pick 1 (sometimes we have added the 7th “blank” option that people can fill in).

**TABLE 1:** Case 1. Female Genital Mutilation

Step 1. Case Description You are a medical doctor working for 4 weeks in north-east Africa, at a small countryside clinic where you are in charge. You are aware that female genital mutilation (FGM) is a widespread practice in those areas. One evening a local midwife, who works with you at the clinic, asks whether it would be possible to use the clinic to perform FGM in the evenings. You are aware that the midwife practices FGM herself. She says that performing the operation in the clinic would be beneficial because of hygienic reasons.
Step 2. Options What would you do and how would you justify it? FGM is a well-established cultural practice, and I have no right to be judgmental about it. Although I don’t support the FGM and make my position clear to the midwife, I am only a short-time visitor, and it is not realistic to change these practices so quickly. I allow for the use of facilities. FGM is a human rights violation, and allowing midwives to operate at my clinic would implicate me (and the international organization running the clinic) in those cruel practices. FGM will not be performed in my facilities. I organize a meeting among the local community leaders to discuss FGM. There I will be able to voice my opposition to it, but nevertheless I will declare from the onset that the participants of the meeting will have the final word. I don’t support FGM, but I take the perspective of the potential patient as the most important one. The girl will be mutilated no matter what I say or do, the only thing I can influence is whether the operation will take place in clean facilities or not. I agree to the use of facilities. I ask local medical staff about how and if such queries have been dealt with before and act according to their suggestions.
Step 3. Discussion
Step 4. Value Reflection (Examples) Option 3: You stand up for your principles and believe that *human rights* ought to be respected universally. You are also *loyal* to your organization. At the same time, you might be seen as abandoning the potential patient and violating the *non-maleficence* principle. Option 5: You take the principles of *beneficence, non-maleficence* as the most important ones. As a doctor you have a duty to particular persons. At the same time you give up your right (and perhaps a duty) to stand up for *public health* issues and *human rights* violations.

Once everyone has made up their mind (no discussion at this point), they should simultaneously make their choice public. We have sometimes distributed preprinted cards with a number on them and these can be revealed (or participants can write a number on a piece of paper). The other option is to simply show your preference with the number of raised fingers. That moment of revealing one’s preference constitutes an important learning occasion, because there is always a variety of answers selected (having used this method with hundreds of students and professionals in culturally and geographically diverse locations, three is the minimum variation we have encountered). Experiencing disagreement with your peers, people whom you generally consider as knowledgeable and ethical as yourself, is an important learning objective here. In contrast to the more usual case-based discussion (without prepared options), the disagreement here is straightforwardly manifest, the individual responsibility for making a decision is evident (and sometimes uncomfortable) and always gets the discussion going quickly.

Everyone is then invited to discuss the reasons for their choice (Table 1, Step 3. Discussion) because even the same option is often chosen for different reasons. Another round that would add a team-working element could be an attempt to find a consensus and offer participants the option to change their initial preferences. Having tried this method in a variety of locations internationally, it might be the case that “changing one’s mind” is socially more acceptable in some places than others. Nevertheless, consensus-seeking as well as sometimes living with disagreements regarding ethical challenges are important skills for any teamwork.

Finally, the method includes an analysis of the ethical values involved in each choice. Value Reflection (Table 1, Step 4) outlines some of the values that are at stake for a particular choice. What should become clear from the analysis of these imperfect options, is that each respects certain important values and disrespects others. Good ethical decision-making is aware of these values and how they might conflict, as well as able to articulate why the values have been prioritized in such ways for particular contexts.

### Strengths of the Value-Reflection Case Method

- This method gets the discussion going quickly, because the “options” are already outlined (shortage of time for ethics training is often the case).
- Because there are numbered choices, disagreement becomes evident from the beginning. The peer disagreement itself, being aware that your peers, whom you trust, think differently, is an important learning objective here.
- As there is no “perfect” option, one is forced to make difficult choices. This feeling of anxiety, not being happy with your choice, mimics ethical decision-making in humanitarian situations. It is an engagement with the “inescapable cruelties” intrinsic to humanitarianism^19^ that offers opportunities to emulate the affective and emotional aspects of crisis decision-making and experience some aspects of moral distress.
- It guides the discussion to the level of values and how we might prioritize them. Although values are abstract concepts, once they are identified and mastered, they can be useful in helping people to articulate and justify their choices.
- The method draws attention to both the individual as well as the collective side of decision-making. At first, the focus is on taking personal responsibility for your decision, because one has to make an individual choice to start with, rather than everyone consecutively voicing opinions (and possibly being influenced by previous contributions), there is more taking of personal responsibility. Even if one is willing to give responsibility away, the giving-away has to happen consciously (for example the “listening to the community” options 4 and 6 in Table 1). The realization that even in difficult situations there is room for personal reflection and integrity might help people in not feeling victimized by the circumstances and pushing them to take responsibility. Second, the consensus-building phase underlines the importance of teamwork and acknowledges the relevance of collective practices and relationships.
- Finally, the value reflection phase combines individual micro-level beliefs with higher principles and values to result in acceptable practical solutions.

### Difficulties of the Approach

One of the difficulties with this approach is it takes time and effort to prepare the cases to suit the method. For example, there should be no “easy, correct choice”, otherwise there is little discussion of substance and no emotional unease.

It has been argued that it is better to let everyone come up with their own options; this is how case-based teaching is mostly done. While this would also be appropriate, our experience has shown that the value-reflection method gets to the dilemmas more quickly and often results in a better discussion (because it creates straightforward disagreements).

## CONCLUSION

Ethical decision-making in humanitarian medicine is often stressful, and good ethics teaching should prepare people better for these kinds of contexts by introducing not only ethics principles and guidelines but mimicking the emotional difficulties of tragic choices. Moral dilemmas cannot entirely be solved or un-problematized; the lingering of doubt, the feeling of helplessness and perhaps even of anger, are likely to accompany even the very best ethical decisions in difficult contexts. The value reflection method takes into account the paradoxes of ethical challenges, the complex emotional and rational arguments and beliefs, the individual and collective decision-making levels, the implicit and the explicit values. Through helping to build a foundation for a more comprehensive response, it hopes to help in preparing humanitarian aid workers for the moral distress that they are likely to experience.
